# Conditional ablation of E‐cadherin in the oral epithelium progeny results in tooth anomalies

**DOI:** 10.1002/cre2.612

**Published:** 2022-06-15

**Authors:** Stephanos Kyrkanides, Denise Trochesset, Maria Cordero‐Ricardo, Sabine M. Brouxhon

**Affiliations:** ^1^ Department of Oral Health Science, College of Dentistry University of Kentucky Lexington Kentucky USA; ^2^ Department of Neuroscience (Adjunct), School of Medicine and Dentistry University of Rochester Rochester New York USA; ^3^ Oral and Maxillofacial Pathology, Radiology and Medicine New York University New York New York USA; ^4^ Department of Pediatric Dentistry, Maurice H Kornberg School of Dentistry Temple University Philadelphia Pennsylvania USA; ^5^ Department of Physiology and Biophysics Stony Brook University Stony Brook New York USA

**Keywords:** E‐cadherin, odontogenesis, oral epithelium

## Abstract

**Objectives:**

The objective of this study is to confirm the developmental origin of the enamel organ and evaluate the role of E‐cadherin in tooth development by conditional deletion in the oral epithelium and its enamel organ progeny. K5‐Cre;ROSA26 compound mice were included in this study in order to confirm the oral epithelial origin of the enamel organ, as well as of the action of the K5‐Cre transgene in ablating E‐cadherin in the enamel organ. K5‐Cre;Ecad^fl/fl^ knockout mice were included to evaluate the effects of the conditional E‐cadherin ablation onto tooth development.

**Material and Methods:**

K5‐Cre transgenic mice were crossed into the ROSA26 reporter mouse to trace the cell fate of the oral epithelium and its progeny in vivo. Moreover, K5‐Cre mice were crossed into the Ecad^fl/fl^ mice to produce K5‐Cre;Ecad^fl/fl^ compound mouse, as well as K5‐Cre;Ecad^fl/+^ and Ecad^fl/fl^ littermate controls. These litters were euthanized at postnatal day P2 to study the effects of conditional E‐cadherin ablation in vivo.

**Results:**

The K5‐Cre;ROSA26 compound mouse demonstrated that the origin of the enamel organ and the structures thereof are of oral epithelial origin. Furthermore, using the K5‐Cre;Ecad^fl/fl^ compound mouse, we determined that conditional ablation of E‐cadherin in the oral epithelium, and its progeny, results in dental anomalies involving elongation of the molar root, shrinkage of the pulp space, and alterations of the periapical area, including cementum hyperplasia. The K5‐Cre;Ecad^fl/fl^ mice also displayed a smaller overall stature compared with heterozygotes and wild‐type littermates.

**Conclusions:**

E‐cadherin is important in tooth development, including the formation of enamel, the crown, pulp space, and the roots.

## 1 INTRODUCTION

Cadherins are a family of calcium‐dependent, transmembrane, cell adhesion molecules responsible for cell aggregation, spatial segregation, organization, and communication. Homotypic, cadherin‐mediated cellular sorting is known to modulate the development of complex morphologic structures. E‐cadherin, which is the prototypic member of cadherins, is expressed at very early stages during embryonic development and is essential for establishing apico‐basal polarity, cell sorting, and tissue‐boundary formation during embryonic development, maintaining epithelial survival, and controls epithelial cell proliferation (Fleming et al., 1992; Gumbiner, 2005; Lien et al., 2006). In contrast to E‐cadherin, N‐cadherin first appears during neurulation and plays an essential role in separation of the neural tube from the E‐cadherin‐positive embryonic ectoderm (Hatta et al., 1987). As such, N‐cadherin is normally expressed in specific cell types including neuroepithelium, neurons, and mesenchymal cells, and has been used as a neuroepithelial or mesenchymal marker (Gumbiner, 2005).

Several investigations have focused on the distinct temporal and spatial pattern of Type 1 (E‐)cadherin molecules during these processes (Luo & Radice, 2005). During the cap and bell stages of tooth development, pre‐ameloblasts express E‐cadherin with an apical‐coronal gradient distributed within the proliferating epithelium (Heymann et al., 2002). E‐cadherin is posttranslationally regulated during amelogenesis by enamelysin (MMP‐20) and is involved in the maturation and mobility of ameloblasts during enamel formation (Bartlett et al., 2011; Guan & Bartlett, 2013). Interestingly, E‐cadherin can replace N‐cadherin during amelogenesis while rescuing the tooth phenotype (Guan et al., 2014). N‐cadherin has a reciprocal expression pattern in the pre‐ameloblasts and is expressed in the mesenchymal precursors of the dentin and pulp tissues (Heymann et al., 2002). This distribution highlights the concept that secretory ameloblasts, as differentiated epithelial cells within the enamel organ, have the ability to produce extracellular matrix, which is contradictory to the classification and properties of the epithelium.

The purpose of the present study was to confirm the oral epithelial origin of the enamel organ and to determine the role of E‐cadherin in enamel development by conditionally ablating the E‐cadherin gene in the oral epithelium and its progeny in vivo.

## 2 MATERIALS AND METHODS

### 2.1 Animal and tissue preparation

All animal‐related work herein was reviewed and approved by the Institutional Committee on Animal Resources at Stony Brook University, before the pursuit of any pertinent research experiments for the necessary protection of animals in research (there were no human research subjects and therefore no informed consent was necessary to be included in this study). The K5‐Cre transgenic mice (Ramirez et al., 1994, 2004) were kindly donated by Dr. Duski Ilic (University of California at San Francisco, USA). The ROSA26 (R26R) conditional reporter mouse model (Soriano, 1999) was purchased from The Jackson Laboratory (Bar Harbor, ME, USA). The Ecad^fl/fl^ and Ecad^+/−^ mice were purchased from The Jackson Laboratory. Genotyping was accomplished by polymerase chain reaction for K5‐Cre as previously described (Ramirez et al., 2004) and for the Ecad mice as described by the vendor.

For the generation of K5‐Cre^+/−^;R26R^+/−^ mice, male K5‐Cre^+/−^ and female R26R^+/+^ mice were bred: 25% of the offspring contained the desired genotype and 25% were controls. A total of two K5‐Cre^+/−^;R26R^+/−^ and two K5‐Cre^−/−^;R26R^+/−^ were used in this study out of one litter. To delete E‐cadherin conditionally in epithelial cells and their progeny, we first mated male K5‐Cre^+/−^ with female Ecad^fl/−^ mice (produced by breeding Ecad^fl/fl^ × Ecad^+/−^ mice). The desired K5‐Cre^+/−^;Ecad^fl/−^ compound mouse, however, was never obtained due to apparent embryonic lethality. Instead, we pursued the mating of male K5‐Cre^+/−^ with female Ecad^fl/fl^ mice so as to generate the K5‐Cre^+/−^;Ecad^fl/fl^ compound mice, which displayed dental anomalies, and were included as part of this study. A total of four K5‐Cre^+/−^;Ecad^fl/fl^ and four K5‐Cre^−/−^;Ecad^fl/fl^ were included in our study originating from two litters.

Pups were euthanized at postnatal day P2 using carbon dioxide gas followed by decapitation. Young adult mice were killed at 4 weeks of age by the same method. The mouse heads were fixed in 10% formalin by immersion over 72 h at 4°C, followed by immersion in a cryoprotective solution (30% sucrose in phosphate‐buffered saline (PBS) at 4°C for 48 h.

### 2.2 Histochemistry

Mouse heads were harvested and sectioned on a cryostat in 20 µm frontal sections collected on poly‐l‐lysine precoated slides. The slides were allowed to dry overnight at room temperature before placement in a −20°C freezer for later use. X‐gal histochemistry was employed for the detection of the reporter gene β‐galactosidase (lacZ) as follows: The sections were rinsed twice for 15 min in 100 mM PBS, 2 mM MgCl_2_, 0.01% sodium deoxycholate, and 0.02% NP‐40 solution. The tissue sections were then incubated overnight in 50 mM potassium ferricyanide, 50 mM potassium ferrocyanide, 1 M MgCl_2_, and 50 mg/ml X‐gal (pH 7.8) at 30°C. The next day, slides were then rinsed twice in 0.15 M PBS, postfixed in 10% buffered formalin for 15 min, and subsequently rinsed with deionized distilled water. The tissue sections were later dehydrated by immersion in ethanol solutions and cleared in xylene. For detection of bone and cartilage cells, the same processing protocol was followed, with the exception that Alcian Blue/Orange G histochemistry was used as chromagens instead of X‐gal. Tissue slides were subsequently cover‐slipped with DPX mounting medium (Fluka, Neu0Ulm, Switzerland) and examined under a light microscope (BX51 Olympus; Tokyo, Japan). Color microphotographic images were captured with a SPOT RT Color CCD digital camera (Diagnostic Instruments, Sterling Heights, Michigan, USA) attached to a Zeiss BX41 microscope connected to a PC computer.

### 2.3 Immunohistochemistry

Immunohistochemistry was employed for the qualitative detection of E‐ and N‐cadherin in the oral epithelium and the developing molar tooth germs. In brief, the mouse heads were decalcified, processed, and paraffin embedded, and sectioned on a microtome at 10 μm‐thick sections. Consequently, the sections were deparaffinized and rehydrated. Tissue sections were first rinsed in PBS for 60 min, bleached using a 3% H_2_O_2_ in PBS solution, and then washed again in PBS. Sections were blocked with normal goat serum (NGS) (4% NGS in 0.15 M PBS), incubated with agitation overnight in the primary antibody solution containing rabbit anti‐E‐cadherin (1:200 dilution) or N‐cadherin (1:1000 dilution) polyclonal antibodies (Santa Cruz Biotechnology; Santa Cruz CA), 0.5% Triton‐C‐100 (PBS‐Tx), followed by a 30 min blocking step (4% in PBS), and 90 min incubation in the secondary antibody solution containing a goat anti‐rabbit IgG biotin conjugated antibody (1:2500) in the presence of 0.5% Triton‐X (Tx) and 4% NGS in 0.15 M PBS. Sections were next washed in PBS‐Tx for 30 min, followed by a 90 min incubation in avidin–biotin complex solution (ABC kit, Vector Laboratories, Burlingame, CA). Subsequently, the tissue sections were washed in 175 mM sodium acetate buffer solution (pH 7.2) for 30 min. Sections were finally reacted in DAB (3,3’ diaminobenzidine)‐nickel solution in 125 mM sodium acetate buffer solution with 10 mM imidazole (pH 9.3), and washed in PBS for 15 min. The tissues sections were subsequently dehydrated, cleared in graded xylenes, cover‐slipped, and analyzed as described above.

## 3 RESULTS

### 3.1 Oral epithelium cell fate

**Figure 1:**
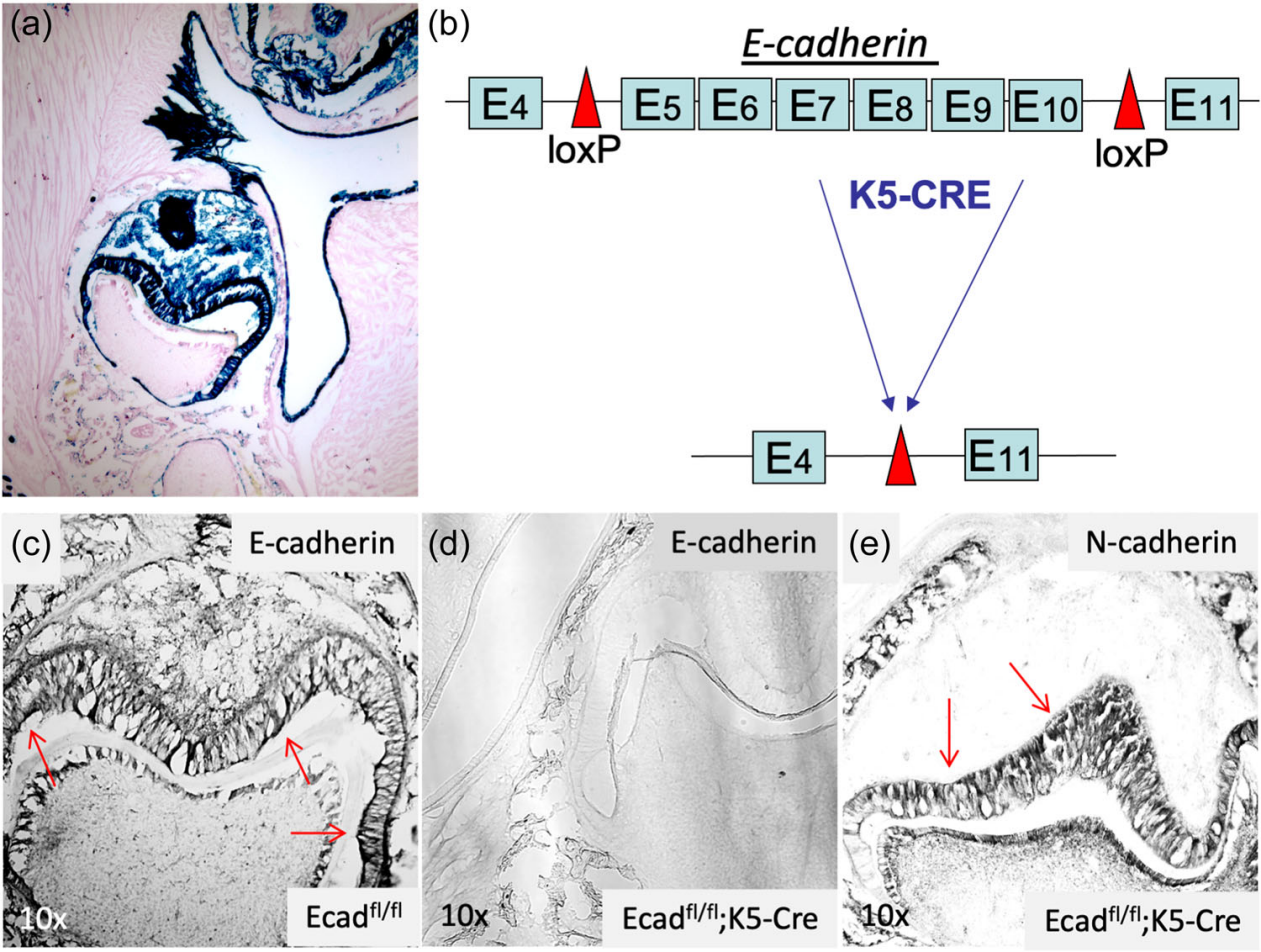
Conditional deletion of E‐cadherin in the oral epithelial progeny. Cytokeratin‐5 (K5)‐driven CRE‐mediated genomic DNA recombination in the mouse was achieved by crossing the K5‐Cre transgenic mouse model with (a) the ROSA26 transgenic mouse model. This strategy commenced activation of the reporter gene β*‐*galactosidase in K5‐expressing tissues, including epithelial keratinocytes (these are skin cells), early in embryonic development, and marked their progeny indelibly, in vivo. Here, the oral epithelium, dental lamina, enamel organ, inner enamel epithelium, and Hertwig's sheath stained positively (blue) with X‐gal histochemistry at postnatal day P2, demonstrating that the enamel organ is of oral epithelium developmental origin. We employed the same strategy (b) to ablate the E‐cadherin gene in the K5‐Cre;Ecad^fl/fl^ compound transgenic mouse, where CRE‐mediated DNA recombination directed by the loxP sites inserted into the gene locus, effectively ablating the E‐cadherin gene by the removal of exons E_6_–E_10_, in vivo. We employed immunohistochemistry to confirm (c) the expression of E‐cadherin in Ecad^fl/fl^ wild‐type littermates (black stain) and (d) the absence of E‐cadherin in the K5‐Cre;Ecad^fl/fl^ experimental mouse littermates. (e) Detection of N‐cadherin, which is known to also be expressed in the tooth bud, was included as a positive control in the K5‐Cre;Ecad^fl/fl^ mouse.

We first traced the fate of the oral epithelium and its progeny in K5‐Cre;ROSA26 compound mice. Expression of the reporter gene β‐galactosidase indelibly marking the oral epithelium progeny was detected by X‐gal blue staining in mice at postnatal day P2 (Figure 1a). We observed that the oral epithelium, dental lamina, and the enamel organ, including the inner enamel epithelium and the Hertwig's root sheath originated from the oral epithelium.

### 3.2 Cell‐fate tracing of oral epithelium progeny in K5‐Cre;ROSA26 mice

Cytokeratin‐5 (K5)‐driven CRE‐mediated genomic DNA recombination in the mouse was achieved by crossing the K5‐Cre transgenic mouse model with the ROSA26 transgenic mouse model. In this model, oral epithelium, dental lamina, enamel organ, inner enamel epithelium, and Hertwig's sheath stained positively (blue) with X‐gal histochemistry at postnatal day P2, demonstrating that the enamel organ is of oral epithelial developmental origin (Figure 1a). To conditionally ablate E‐cadherin in the oral epithelium, and its progeny, we crossed the K5‐Cre transgenic mouse with the Ecad^fl/fl^ mouse, as demonstrated in the schematic (Figure 1b). Wild‐type littermates (Ecad^fl/fl^) demonstrated E‐cadherin expression throughout the enamel organ, as detected by immunohistochemistry (Figure 1c). In contrast, K5‐Cre;Ecad^fl/fl^ compound mice exhibited a near lack of E‐cadherin expression, especially in the oral epithelium, as depicted by immunohistochemical staining for E‐cadherin after ablation (Figure 1d). As a control, we stained tissues from K5‐Cre;Ecad^fl/fl^ mice for N‐cadherin, which revealed positive immunostaining, after E‐cadherin ablation (Figure 1e).

### 3.3 Developmental anomalies in the K5‐Cre;Ecad^fl/fl^ mice

**Figure 2:**
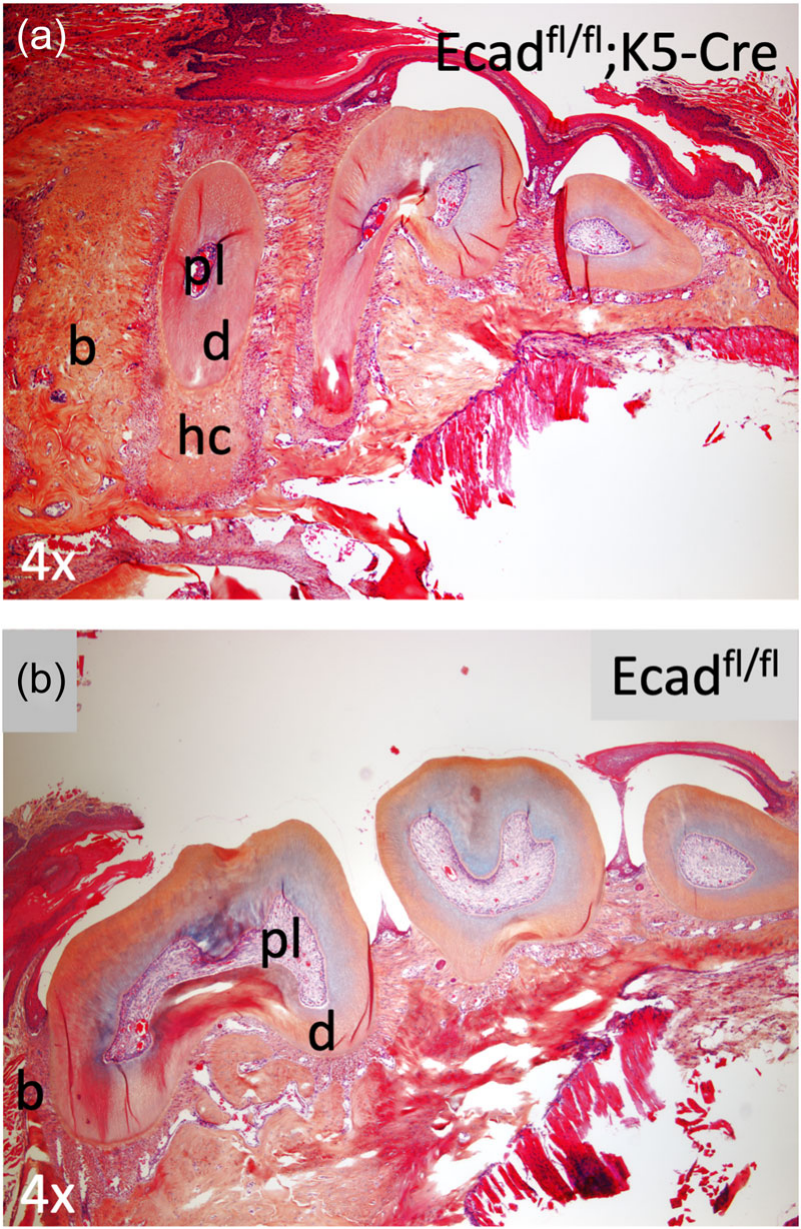
E‐cadherin deletion in the oral epithelium and its progeny affects tooth development. Sections of the mandible harvested from K5‐Cre;Ecad^fl/fl^ experimental mice and Ecad^fl/fl^ wild‐type littermates were histologically examined using Alcian Blue/Orange G histochemistry. (a) Mandibular teeth in the K5‐Cre;Ecad^fl/fl^ mice displayed interestingly an abnormal morphology comprising enlarged cementum within elongated roots and an almost absent pulp. (b) Control Ecad^fl/fl^ mice displayed normal tooth shape and structure with vivid pulpal space, homogeneous enamel, and dentin thickness.

**Figure 3:**
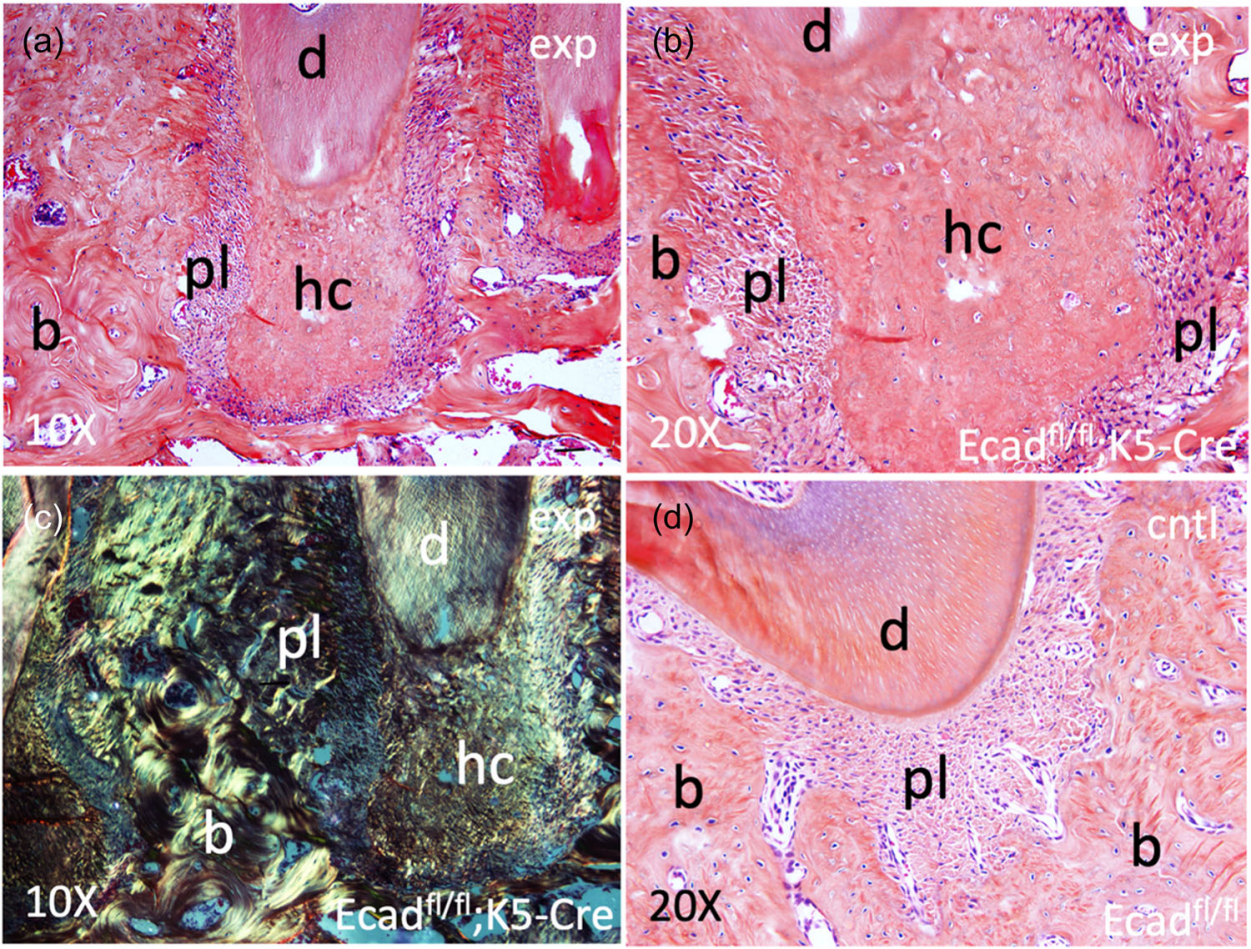
E‐cadherin deletion in oral epithelium and its progeny affects root development. Sections of the mandibular teeth harvested from K5‐Cre;Ecad^fl/fl^ experimental mice and Ecad^fl/fl^ wild‐type littermates were histologically examined using Alcian Blue/Orange G histochemistry. (a) Hypercementosis (hc) was observed apical to the root apex in the K5‐Cre;Ecad^fl/fl^ experimental mice. (b) Larger magnification focused on the area displaying cellular hypercementosis. (d) Comparable area of specimens harvested from Ecad^fl/fl^ wild‐type littermates showing normal histological cytoarchitecture of the involved tissues, including acellular cementum. (c) Polarized filter images captured from (b), confirming distinct architectural structure of hypercementosis in the K5‐Cre;Ecad^fl/fl^ mouse model. b, alveolar bone; d, dentin; pl, periodontal ligament.

**Figure 4:**
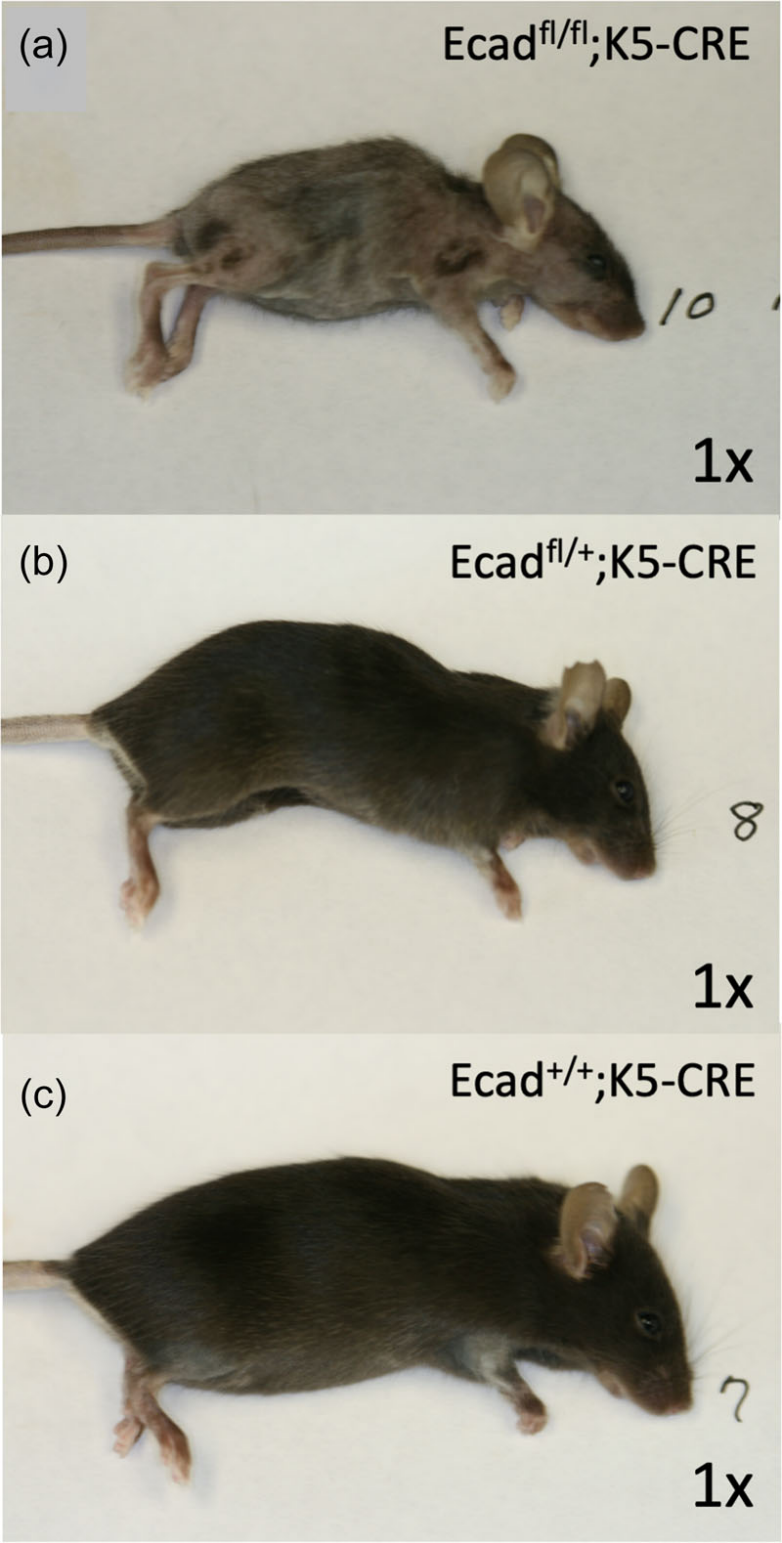
K5‐Cre;Ecad^fl/fl^ mice have short statue. The mating strategy of K5‐Cre with the Ecad^fl/fl^ mouse produced three viable genotypes: K5‐Cre;Ecad^fl/fl^, K5‐Cre;Ecad^fl/+^, and K5‐Cre;Ecad^+/+^. Visual inspection revealed that the K5‐Cre;Ecad^fl/fl^ display a statue shorter than the heterozygote K5‐Cre;Ecad^fl/+^ and wild‐type K5‐Cre;Ecad^+/+^ littermates.

Comparative Alcian Blue/Orange G histology staining on sections harvested from K5‐Cre;Ecad^fl/fl^ and wild‐type littermate mice (Ecad^fl/fl^) reveals the development of dental anomalies resulting from the conditional ablation of E‐cadherin in the oral epithelium and its progeny. Specifically, compared with wild‐type littermate controls, we observed elongation of the molar root, shrinkage of the pulp space, and alteration of the periapical area (Figure 2). Closer evaluation of the premolar periapical region revealed the development of hypercementosis (hc) distal to the root apex (Figure 3a–c). Although cementum is typically noted as lamellated layers of acellular cementum laid down on the root surface, the thickness of which increases over time, the affected (can put designation instead) mice show an accumulation of a product that is attached to the root, has no lamellated layers and demonstrates scattered lacunae containing viable cells within the product. We note that there is an increase in cellularity in the stroma around the affect root and product. Additionally, K5‐Cre;Ecad^fl/fl^ pups exhibited a smaller overall stature and displayed alterations in the tail and nails (lengthened), and elongation of the snout (Figure 4a), compared with heterozygotes (Figure 4b) and wild‐type littermates (Figure 4c).

## 4 DISCUSSION

The goal of this study was to first confirm the oral epithelial origin of the enamel organ, as well as of the action of the K5‐Cre transgene in ablating E‐cadherin in the enamel organ; secondly, to evaluate the effects of the conditional E‐cadherin ablation onto tooth development. To identify the cells wherein E‐cadherin would be ablated based on this strategy, we first studied the cell fate of the oral epithelium in the K5‐Cre;ROSA26 compound mouse during tooth development. Such strategy employing the reporter mouse ROSA26 has been previously employed by Chai et al. (2000) in tracing the fate of neural crest cells in tooth development. Our choice of the transgenic mouse whereby Cre recombinase is driven by the K5 promoter was based on the fact that K5 is expressed ubiquitously in epithelial cells (Ramirez et al., 1994). Therefore, the progeny of oral epithelium in the K5‐Cre;ROSA26 compound mouse would be indelibly marked by the expression of the reporter gene β‐galactosidase and be readily detected by X‐gal histochemistry, therefore allowing for cell‐fate tracing of the oral epithelium progeny (Chai et al., 2000). Our results demonstrate that the dental lamina and the enamel organ developmentally originate from the oral epithelium, along with the cells contained therein, including pre‐ameloblasts and ameloblasts. Although the origin of the enamel organ has been known from descriptive histological studies, (Nanci, 2012), this is the first animal study using molecular biology techniques to conclusively confirm in vivo the epithelial origin of the dental lamina and enamel organ.

**Figure 5:**
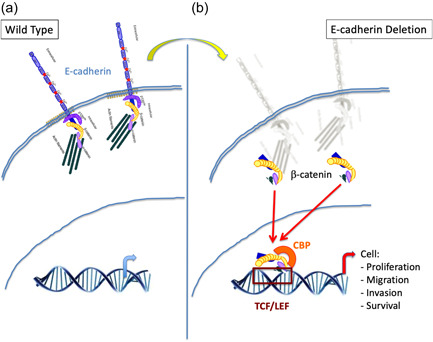
Schematic summary: β‐catenin‐mediated gene activation by loss of E‐cadherin. In normal conditions, such as in wild‐type mice, (a) E‐cadherin is found transversing the cell membrane of epithelial cells, whereby the intracellular signaling factor β‐catenin is anchored on its cytoplasmic domain. Conversely, in situation where E‐cadherin is not present, (b) such as in the K5‐Cre;Ecad^fl/fl^ mouse model where it has been deleted, β‐catenin is free to enter from the cytoplasm into the cell nucleus, where it can activate numerous genes that promote cell proliferation, migration, invasion, and survival.

The periapical tissue observed in the K5‐Cre;Ecad^fl/fl^ mice resulted from the ablation of E‐cadherin in the oral epithelium and its progeny. E‐cadherin is posttranslationally regulated during amelogenesis by enamelysin (MMP‐20; Bartlett et al., 2011; Guan & Bartlett, 2013; Guan et al., 2014). E‐cadherin through its cytoplasmic domain binds to β‐catenin (Figure 5), as well as to other actin‐affiliated proteins (Huber & Weis, 2001). When E‐cadherin is shed from the cell surface (e.g., via the action of MMP‐20 [Bartlett et al., 2011; Guan & Bartlett, 2013; Guan et al., 2014]), β‐catenin is mobilized intracellularly and enters into the nucleus to form a complex with the LEF/TCF family, resulting in transcriptional activation as well as cellular proliferation, migration, and invasion (Huber & Weis, 2001; Moon et al., 2004). Furthermore, β‐catenin promotes continuous tooth development when activated in the embryos, whereas β‐catenin ablation induces enamel malformation (Guan et al., 2016; Jarvinen et al., 2006; Liu et al., 2010). β‐Catenin was also shown to be critical for ameloblast migration in ALC cells, and when treated with a β‐catenin inhibitor, prevented migration and significant upregulated E‐cadherin (Guan et al., 2016). β‐Catenin deletion in mice causes enamel malformations in vivo, in part, due to a lack of ameloblast movement necessary to form the decussating enamel rod structure (disruption of ameloblast differentiation and/or mobility; Guan et al., 2016), whereas β‐catenin stabilization gives rise to multiple teeth in vivo (Jarvinen et al., 2006; Liu et al., 2010).

Similarly, we observed hyperplasia apical to the roots of teeth in the K5‐Cre;Ecad^fl/fl^ mice. E‐cadherin deletion, as discussed above, is linked to mobilization of β‐catenin and induction of genes the promote cell proliferation, as well as invasion. Under this scenario, the histological appearance of hypercementosis is the result of E‐cadherin ablation in the population of cells responsible for the formation of cementum. This cementum‐like product does not have the appearance of the native bone and is not in continuity with the bone of the jaw. This appearance has some similarity to the histopathologic findings seen with cementoblastoma, a benign neoplasm of cementoblasts, which produces cementum‐like tissue connected to the tooth root. Cementoblastomas show a deposition of cementum‐like product, often with lacunae, in association with a proliferation of plump osteoblasts. This cellular component of plump cementoblasts aggregates is not seen in our tissue. Rather the cells in our tissue look very similar to the connective tissue stromal cells, just in a more compact pattern, and this is why we identify the product as cementum‐like rather than saying there are cementoblastomas at the roots of all the teeth. Clinically, generalized hypercementosis was described in a report of a patient presenting with a condition similar to our observations in the K5‐Cre;Ecad^fl/fl^ mice based on the radiographic features described therein, lacking, however, any analysis of the genetic etiology of the clinical condition (Seed & Nixon, 2004). However, it is possible that disruption in E‐cadherin levels, either at the transcription or posttranslational levels, may be the underlying etiology of clinical hypercementosis. In the future, genetic studies may be pursued to identify the role of E‐cadherin in clinical hypercementosis and similar hypertrophic conditions.

## AUTHOR CONTRIBUTIONS

Stephanos Kyrkanides contributed to the experimental design, execution of experiments, collection of data, interpretation of data, and composition of the manuscript. Denise Trochesset contributed to the collection of data, interpretation of data, and composition of the manuscript. Maria Cordero‐Ricardo contributed to the composition of the manuscript. Sabine M. Brouxhon contributed to the the experimental design, execution of experiments, collection of data, interpretation of data, and composition of the manuscript.

## CONFLICT OF INTEREST

The authors declare no conflict of interest.

## Data Availability

Data will be available upon request.
